# Homologous Strategy to Construct High-Performance Coupling Electrodes for Advanced Potassium-Ion Hybrid Capacitors

**DOI:** 10.1007/s40820-020-00524-z

**Published:** 2020-10-30

**Authors:** Ying Xu, Jiafeng Ruan, Yuepeng Pang, Hao Sun, Chu Liang, Haiwen Li, Junhe Yang, Shiyou Zheng

**Affiliations:** 1 School of Materials Science and Engineering, University of Shanghai for Science and Technology, Shanghai, 200093 People’s Republic of China; 2 College of Materials Science and Engineering, Zhejiang University of Technology, Hangzhou, 310014 People’s Republic of China; 3 International Research Center for Hydrogen Energy, Kyushu University, Fukuoka, 819-0395 Japan

**Keywords:** Carbon fibers, Potassium-ion capacitor, Alkali-ion battery

## Abstract

Potassium-ion hybrid capacitors (PIHCs) have been considered as promising potentials in mid- to large-scale storage system applications owing to their high energy and power density. However, the process involving the intercalation of K^+^ into the carbonaceous anode is a sluggish reaction, while the adsorption of anions onto the cathode surface is relatively faster, resulting in an inability to exploit the advantage of high energy. To achieve a high-performance PIHC, it is critical to promote the K^+^ insertion/desertion in anodic materials and design suitable cathodic materials matching the anodes. In this study, we propose a facile “homologous strategy” to construct suitable anode and cathode for high-performance PIHCs, that is, unique multichannel carbon fiber (MCCF)-based anode and cathode materials are firstly prepared by electrospinning, and then followed by sulfur doping and KOH activation treatment, respectively. Owing to a multichannel structure with a large interlayer spacing for introducing S in the sulfur-doped multichannel carbon fiber (S-MCCF) composite, it presents high capacity, super rate capability, and long cycle stability as an anode in potassium-ion cells. The cathode composite of activated multichannel carbon fiber (aMCCF) has a considerably high specific surface area of 1445 m^2^ g^−1^ and exhibits outstanding capacitive performance. In particular, benefiting from advantages of the fabricated S-MCCF anode and aMCCF cathode by homologous strategy, PIHCs assembled with the unique MCCF-based anode and cathode show outstanding electrochemical performance, which can deliver high energy and power densities (100 Wh kg^−1^ at 200 W kg^−1^, and 58.3 Wh kg^−1^ at 10,000 W kg^−1^) and simultaneously exhibit superior cycling stability (90% capacity retention over 7000 cycles at 1.0 A g^−1^). The excellent electrochemical performance of the MCCF-based composites for PIHC electrodes combined with their simple construction renders such materials attractive for further in-depth investigations of alkali-ion battery and capacitor applications.

**Electronic supplementary material:**

The online version of this article (10.1007/s40820-020-00524-z) contains supplementary material, which is available to authorized users.

## Highlights

- A novel and facile homologous strategy is proposed to construct unique multichannel carbon fiber (MCCF)-based electrode materials for potassium-ion hybrid capacitors.
- The S-MCCF anodes present high capacity, super rate capability, and long cycle stability in potassium-ion half-cells, and the aMCCF cathodes have a high specific surface area of 1445 m^2^ g^−1^ and exhibit outstanding capacitive performance.
- The fabricated PIHC (*m*_anode_:*m*_cathode_ = 1:2) devices show high energy and power densities together with excellent cycling stability.

**Electronic supplementary material:**

The online version of this article (10.1007/s40820-020-00524-z) contains supplementary material, which is available to authorized users.

## Graphical abstract

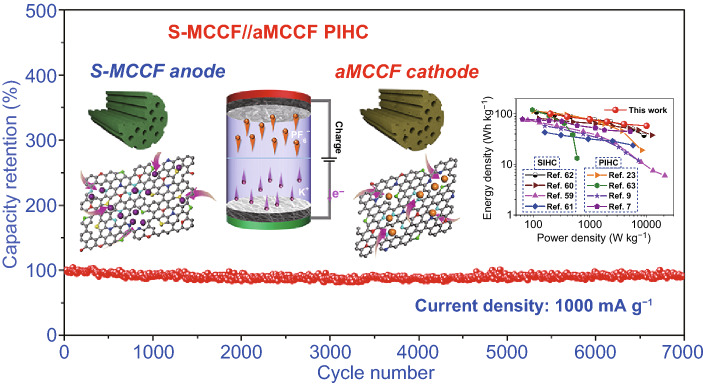

## Introduction

Renewable energy, including wind, solar, and tidal, is currently being developed due to severe environmental pollution and rapid consumption of fossil fuels. In practical applications, electrochemical energy storage systems (such as advanced capacitors and batteries) are used as the main energy media [1–3]. Among these, potassium-ion batteries (PIBs) have gained significant attention and have been widely investigated owing to the abundant and widespread availability of K on earth (K: 2.09 wt%, 17,000 ppm) and its low cost [4–6]. However, PIBs exhibit a relatively lower power density compared with lithium-ion or sodium-ion batteries, which cannot fulfill the mid- to large-scale energy storage system demands, particularly in fast charging/discharging equipment. Potassium-ion hybrid capacitors (PIHCs) are considered promising alternatives, as they combine the merits of the high energy density of PIBs and high power density of supercapacitors (SCs) combined with long cycle life [7, 8]. In practical applications, the properties of the constituent cathode and anode materials are a critical factor governing the operation of electrochemical devices.

In the case of PIHCs, the rate performance and cycling stability are mainly determined by the anode materials. Therefore, various strategies have been explored to construct anodes for PIHCs, for example, the hydrothermal method was used to prepare K_2_Ti_6_O_13_ microscaffolds [9], electrospraying technology was used to fabricate Ca_0.5_Ti_2_(PO_4_)_3_@C microspheres [7], chemical vapor deposition (CVD) technology was applied to prepare carbon nanosheets (CNSs) with disordered, defective, large interlayer spacing, and oxygen-rich features [10] and a combustion method was used to synthesize onion-like carbon (OLC) [11]. Among all the anode materials for PIHCs, amorphous carbonaceous materials can be easily prepared and can present excellent cycling stability [5, 12]. However, these materials suffer from a poor rate performance owing to sluggish K^+^ insertion/desertion kinetics. Therefore, extensive research has been performed to surmount these challenges, of which heteroatom doping is considered as the most successful technique [13–15]. The introduction of a heteroatom can not only create active sites for the adsorption of charges, but also enlarge the interlayer spacing of the carbonaceous matrix for the transport of ions [16–18]. For example, Share and Xu prepared nitrogen-doped graphene and nitrogen-doped carbon nanofibers to improve the rate performance of PIBs [19, 20]. Gong introduced a boron-doped graphene used for PIB, exhibiting a large capacity of 564 mAh g^−1^ and good rate performance [21]. Ma et al. [22] fabricated a phosphorus and oxygen dual-doped graphene and demonstrated a high capacity of 474 mAh g^−1^ at 50 mA g^−1^ after 50 cycles. Qiu and Yang reported that high-performance PIHCs can be achieved with nitrogen-doped hierarchical porous hollow carbon spheres and 3D nitrogen-doped framework carbon as battery-type anode materials, respectively [23, 24]. S-doping is also regarded as an effective technique because the S atom has a relatively larger covalent radius (S 102 pm vs. C 77 pm vs. N 75 pm vs. O 73 pm), which can greatly enlarge the interlayer spacing of carbonaceous materials [25, 26]. Recently, S-doped carbon materials such as S-doped reduced graphene oxide sponges [27], S/O co-doped porous hard carbon microspheres [28], and S-grafted hollow carbon spheres [29], sulfur-doped bamboo charcoal [30] have been extensively explored as anodes for PIBs. Although these electrode materials have demonstrated an encouraging specific capacity, the rate capability, and cycle life do not yet satisfy the demands of real applications. Meanwhile, to fabricate high-performance PIHCs, developing a suitable cathodic material that matches the anode is also a key research challenge. The previous reports show that dual-carbon devices have many advantages, such as low manufacturing cost, good environmental friendliness, and high safety. Besides, the dual-carbon devices are dual-ion energy storage systems, which means the electrochemical properties can be selectively adjusted to meet different application requirements [31, 32]. On the other hand, Chen et al. reported that the three-dimensional (3D) nitrogen-doped framework carbon (3DNFC) anode and cathode materials derived from a single precursor-ethylene diamine tetraacetic acid tetrasodium enhanced the cycling stability of the PIHCs owing to the chemical stability of two electrodes together with *N*-doping [24]. Motivated by these results, ideal PIHCs cathodic materials should have similar chemical stability with anode except for suitable architecture and heteroatom doping. Therefore, the development of advanced cathode materials and design of reasonable electrode structures for PIHCs, though highly desirable, remains considerably challenging.

In this study, we propose a facile “homologous strategy” to fabricate a sulfur-doped multichannel carbon fiber (S-MCCF) anode and activated multichannel carbon fiber (aMCCF) cathode for PIHC. In particular, the PIHC coupling with the S-MCCF and aMCCF achieves high energy and power densities along with super cycling stability. The superior electrochemical properties of the PIHC electrodes based on MCCF composites combined with their simple construction render such materials attractive for further in-depth investigations of alkali-ion battery and capacitor applications.

## Experimental Section

### Preparation of S-MCCF

First, PAN/PMMA virgin fibers were prepared as our previous study reported [33]: briefly, feeding 8 wt% PAN (Mw = 150,000) and 16 wt% PMMA (Mw = 120,000) into *N*,*N*-dimethyl formamide (DMF) solvent and stirring until totally dissolved. Then, a single-solution electrospinning was carried out with the parameter of electrospinning set as follows: high voltage of 18 kV; flow rate of 2 mL h^−1^. The virgin fibers were stabilized at 270 °C in a muffle furnace and kept for 1 h. Then, the stabilized PAN/PMMA fibers were ground into powder and placed on one side of the crucible, and the sulfur powder (PAN/PMMA:S = 1:2, m:m) was placed on the other side. The carbonization process for S-MCCF was conducted at 800 °C in an inert atmosphere of N_2_ and kept for 2 h. As the previous reports show PAN (sheath) will be converted to amorphous carbon while PMMA (core) evaporate leaving a channel in PAN-carbon during the carbonization process because of the different properties of PAN and PMMA [34–36]. In our experiment, multichannels in PAN-carbon fibers formed during the heating process because of the homogeneous mixing of PAN and PMMA, and S atoms doping in the MCCF happened at the same time [37, 38].

### Preparation of aMCCF

The aMCCF material was fabricated by a typical activation process with KOH as activator. First, the stabilized PAN/PMMA fibers were carbonized at 800 °C in N_2_ and kept for 2 h to obtain MCCF. Then, the MCCF material was ground into powder and added into 1.0 M KOH solution with a MCCF:KOH mass of 1:2 and stirred for 24 h; then, the mixed solution was dried to remove the water. After that, the dried mixture of MCCF/KOH was activated at 800 °C in N_2_ atmosphere for 0.5 h. During this process, the reaction of “2 KOH + 2C → 2K + 2CO↑ + H_2_↑” happened, leaving abundant pores in MCCF matrix [39]. Then, 1.0 M HCl aqueous solution was used to neutralize the extra KOH activation agent and react with K in the obtained product. Afterward, the products were washed with deionized water until PH = 7 and dried in a vaccum oven at 80 °C for overnight to obtain the aMCCF with large specific surface area.

### Materials Characterization

A FESEM and a HRTEM were used to observe the morphologies and microstructures of S-MCCF and aMCCF. The surface chemical species of S-MCCF were investigated by XPS. An XRD equipped with a Cu Kα radiation was used to obtain the XRD patterns, and the tests were carried out in the 2*θ* range from 10° to 90° at a scan rate of 5° min^−1^. A LabRAM HR Evolution spectrograph with a He/Ne laser was applied to record the Raman spectra between 600 and 2400 cm^−1^. The pore size distributions and the Brunauer–Emmett–Teller (BET) surface area for S-MCCF and aMCCF were measured based on the nitrogen adsorption/desorption isotherms tested with a surface area and porosity analyzer. The content of S element in S-MCCF material was tested with an Element Analyzer (vario Micro cube).

### Assembling of Potassium-Ion Half-cells

The S-MCCF anode and aMCCF cathode were prepared by a typical slurry-casting method. To elaborate, the S-MCCF anodes were fabricated by homogenously mixing S-MCCF, black carbon, and polyvinylidene fluoride (PVDF) (8:1:1, m:m:m) in *N*-methyl-2-pyrrolidone (NMP) and casted on a Cu foil. The aMCCF cathodes were prepared by homogenously mixing aMCCF, black carbon, and carboxy methylated cellulose (CMC) (8:1:1, m:m:m) in a proper amount of mixture solvent of ethanol and deionized water (ethanol:deionized water = 1:3, m:m) and casting on an Al foil. These electrodes were vacuum-dried overnight at 80 °C before assembling the CR2032-type coin cell in an argon-filled glove box. The potassium-ion half-cells were assembled using K metal foil as counter electrode and glass fiber (GF/D, Whatman) as separator. The electrolyte used was 0.8 M KPF6 in a mixture solvent of ethylene carbonate (EC) and diethyl carbonate (DEC) (1:1, v/v).

### Assembling of PIHC Devices

Before constructing the PIHC devices, S-MCCFs were pre-activated by charging/discharging at a current density of 0.1 A g^−1^ for 10 cycles and then discharged to 0.01 V (vs K/K^+^) in a potassium-ion half-cell. By disassembling this potassium-ion half-cell, pre-activated S-MCCF can be obtained. The PIHC devices were assembled with the pre-activated S-MCCF as anode and aMCCF as cathode. The mass loading of the total active material is about 2.4 mg, and the mass ratios of S-MCCF to aMCCF were fixed at 1:1, 1:2, and 1:3, respectively. Besides, PIHCs with S-MCCF to MCCF (without KOH activation) mass ratio of 1:2 were also assembled as a comparison.

### Electrochemical Measurements

A Land CT2001A battery testing system was applied to test the galvanostatic charge/discharge electrochemical performances at 25 °C. Cyclic voltammetry (CV) was recorded with the electrochemical workstations (CHI 660E). The voltage range for S-MCCF, aMCCF, and PIHC were 0.01–3, 1.5–4, and 0.01–4 V, respectively. The capacitances of potassium-ion half-cells and PIHCs were calculated based on GCD curves with Eq. 1:

1 $$C \, = \, I \, \times \Delta t/\left[ {\left( {V_{\hbox{max} } - V_{\hbox{min} } } \right) \, \times \, m} \right] \, \left( {{\text{F}}\;{\text{g}}^{ - 1} } \right)$$

where *I* presents the discharge current (A), Δ*t* refers to the discharge time (s), *V*_max_ and *V*_min_ are voltages at the beginning and end of the discharge (*V*), and *m* is equal to the mass of the corresponding active electrode.

The energy density (*E*) and power density (*P*) of PIHCs were calculated based on the cell voltage (*V*), the specific capacitance (*C*), and the discharging time (*t*) with the following equations:

2 $$E \, = \, CV^{2} /7.2 \, \left( {{\text{Wh}}\;{\text{kg}}^{ - 1} } \right)$$

3 $$P \, = \, E/t \times 3600 \, \left( {{\text{W}}\;{\text{kg}}^{ - 1} } \right)$$

## Results and Discussion

### Preparation and Characterization of Electrodes

**Fig. 1:**
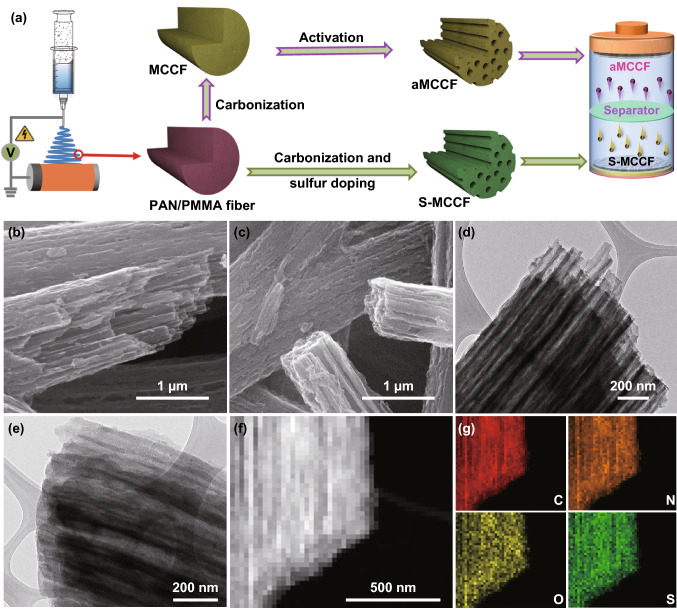
**a** Schematic of the process used to prepare the S-MCCF anode and aMCCF cathode, and the construction of the S-MCCF//aMCCF dual-carbon PIHC. Characterization of the S-MCCF anode and aMCCF cathode: SEM images of the S-MCCF (**b**) and aMCCF (**c**), TEM images of the S-MCCF (**d**) and aMCCF (**e**), **f** TEM image and **g** corresponding element distribution in a fiber of the S-MCCF

The preparation process of the S-MCCF anode and aMCCF cathode and the assembly of the S-MCCF//aMCCF dual-carbon PIHCs are schematically illustrated in Fig. 1a. First, the polyacrylonitrile/polymethyl methacrylate (PAN/PMMA) fibers were prepared by electrospinning the mixed solution of PAN and PMMA (PAN:PMMA = 1:2, m:m). The S-MCCFs were then fabricated by combining the carbonization process of the PAN/PMMA fibers with synchronous sulfur-doping techniques, while aMCCFs were obtained via KOH activation of the MCCF and washing treatment. Finally, the above-prepared S-MCCF and aMCCF were used as anode and cathode materials when assembling the dual-carbon PIHCs. The material preparation process is detailed in the experimental section.

Field emission scanning electron microscope (FESEM) and high-resolution transmission electron microscope (HRTEM) measurements were performed to investigate the microstructure of the S-MCCF and aMCCF samples. Compared with the MCCF (Fig. S1), the S-MCCF (Fig. 1b) and aMCCF samples (Fig. 1c) appear similar, demonstrating that neither S-doping nor KOH activation changed the morphology of the MCCF matrix. The TEM results further confirmed the integrity of the multichannel structure of the prepared MCCF-based materials (Fig. 1d, e). The stable structures of both the anode and cathode materials might enable the development of PIHCs with a long cycle performance. In addition, the corresponding energy dispersive X-ray spectrometry (EDS) elemental maps of the S-MCCF are shown in Fig. 1g. The N and O maps followed the multichannel structure. It can also be seen that the S map shows good agreement with the C map, indicating that S was uniformly doped in the S-MCCF. Further, the content of S element in S-MCCF was tested, and the results showed that the content of S in the material was 6.4%.

**Fig. 2:**
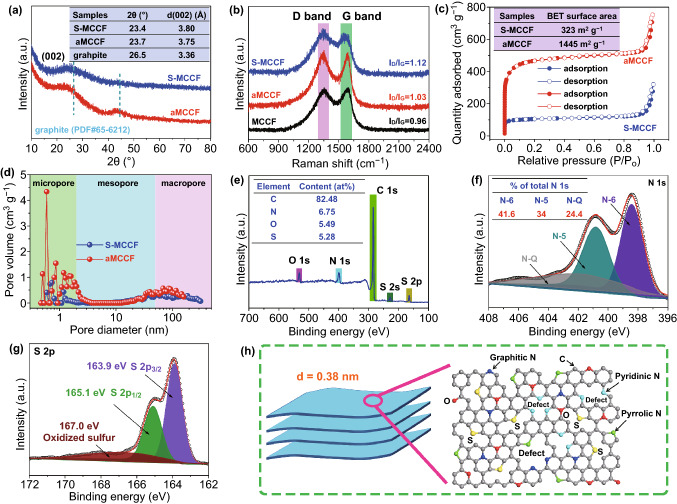
Characterization of S-MCCF and aMCCF: **a** XRD patterns, **b** Raman spectrum, **c** N_2_ adsorption–desorption isothermal curves, **d** pore size distributions, **e** XPS survey spectra corresponding to the S-MCCF and the high-resolution spectrum of **f** N 1 s and **g** S 2p. **h** Schematic of the atomic structure of the S-MCCF

The phase structures of the S-MCCF and aMCCF were characterized using X-ray diffraction (XRD), as displayed in Fig. 2a. The XRD pattern 3 s of the S-MCCF and aMCCF samples show only one broad peak corresponding to (002) diffraction (23.4° for S-MCCF and 23.7° for aMCCF), suggesting the presence of amorphous carbon materials. According to Bragg’s law, the interlayer distances of the S-MCCF and aMCCF are 3.8 Å (in consistent with the result of TEM shown in Fig. S2) and 3.75 Å, respectively, which are marginally higher than that of graphite (3.36 Å, Powder diffraction file No. 65–6212). The enlarged interlayer distance material can be ascribed to the S-doping for the S-MCCF and the KOH activation for the aMCCF. The Raman spectra of the S-MCCF and aMCCF samples are shown in Fig. 2b. The intensity ratio of the D- to G- bands (*I*_*D*_*/I*_*G*_) of the S-MCCF (*I*_*D*_*/I*_*G*_= 1.12) is greater than that of the aMCCF (*I*_*D*_*/I*_*G*_= 1.03), indicating more local defects and disorders in the S-MCCF.

Figure 2c, d reveals the N_2_ adsorption–desorption isothermal curves and pore size distributions of the S-MCCF and aMCCF. It can be seen that both the S-MCCF and aMCCF have hierarchical pores, with most being micro- and macro-pores. The macro-pores in these two materials are related to the multichannel structure. The small number of micropores in the S-MCCF is associated with defects arising from the material preparation processes of high-temperature carbonization and S-doping. In the case of aMCCF, a large number of micropores are mainly derived from KOH activation. The reaction between KOH and C would produce K together with CO and H_2_. The evolution of the CO and H_2_ gases should form many micropores, significantly increasing the specific surface area of the aMCCF (1445 m^2^ g^−1^). Clearly, the specific surface area of the aMCCF (1445 m^2^ g^−1^) is larger than that of activated carbon (AC) (1279 m^2^ g^−1^, shown in Fig. S3), indicating a higher capacitance of aMCCF cathode. Furthermore, prior to being flushed by HCl and water, the formed K atoms tend to enlarge the interlayer spacing of aMCCF, which was verified by XRD. Such a unique structure with an expanded interlayer can potentially promote the insertion/desertion of K^+^.

The surface chemical states of S-MCCF were analyzed using X-ray photoelectron spectroscopy (XPS), as shown in Fig. 2e–g. Figure 2e presents the survey scan that clearly shows the binding energy spectra of S 2s and S 2p, suggesting that the S atoms were doped successfully. Furthermore, N 1s and O 1s can also be seen, which can help improve the specific capacity of the S-MCCF electrode. To elaborate, a small number of O atoms can increase the wettability of carbon and therefore improve the effective surface area available for charge storage [40, 41]. With respect to the *N* atoms, the high-resolution spectrum (Fig. 2f) exhibits three peaks at 398.4 eV (pyridinic N, N-6), 400.85 eV (pyrrolic N, N-5), and 401.6 eV (graphitic N, N-Q). Among these peaks, N-6 and N-5 are related to the defects and active sites, which facilitate the storage of more K^+^ [42]; N-Q is favorable for the electronic conductivity of the S-MCCF and consequently enhances the rate capability [40, 42]. The high-resolution spectrum of S 2p (Fig. 2g) shows that S 2p is deconvoluted into three peaks, where the major peaks of 163.9 and 165.1 eV correspond to S 2p_3/2_ and S 2p_1/2_, respectively, of the –C–S–C– bond, being equivalent to thiophene-type sulfur [25, 28], while the third low-intensity peak at 167.0 eV is consistent with oxidized sulfur [43]. Based on the above analysis, a schematic of the unique structure of S-MCCF can be obtained, as shown in Fig. 2h.

### Electrochemical Performance and Kinetics Analysis of S-MCCF Electrode

**Fig. 3:**
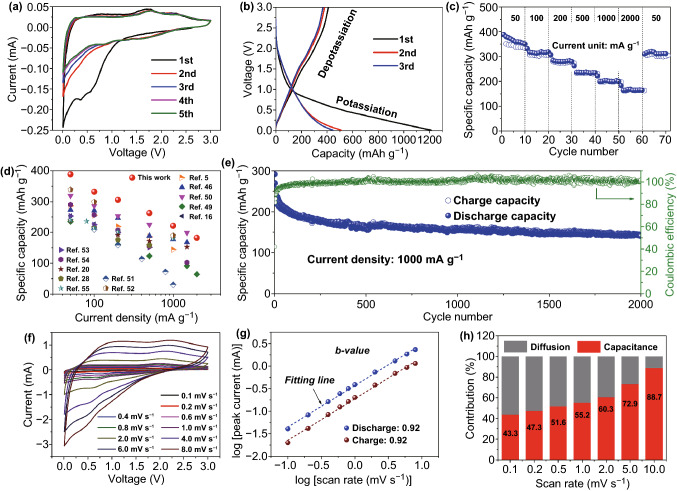
Electrochemical properties of the S-MCCF in PIBs: **a** cyclic voltammetry curves at a scan rate of 0.1 mV s^−1^, **b** galvanostatic charge/discharge profiles of the first three cycles at 50 mA g^−1^, **c** rate performance tested at various current densities (excluding the data of the first cycle at a small current density of 50 mA g^−1^), **d** rate capability of the S-MCCF compared with that of the other carbonous electrodes, and **e** long-term cycling property at 1000 mA g^−1^ (excluding the data of the first cycle). Electrochemical dynamics analysis of the K^+^ storage mechanism of the S-MCCF: **f** cyclic voltammetry curves at various scan rates, **g** determination of the *b*-value, and **h** contribution ratios of the capacitance and diffusion capacities at scan rates ranging from 0.1 to 10 mV s^−1^

The electrochemical performances of the S-MCCF electrode were examined in potassium-ion half-cells. Figure 3a displays the initial five cyclic voltammetry (CV) curves of S-MCCF from 0.01 to 3 V (vs K/K^+^) at the scanning rate of 0.1 mV s^−1^. In the first discharge cycle, a broad peak beginning at 0.9 V and a weak peak at 0.57 V can be observed. This broad peak can be attributed to the irreversible reaction of K^+^ with the functional groups in the S-MCCF surface [27], while the low-intensity peak at 0.57 V is caused by the formation of a solid electrolyte interface (SEI) [11]. Furthermore, a cathodic peak centers at 0.37 V also appears in the first discharge, reflecting the intercalation of K^+^ into the S-MCCF [44]. The oxidation peak at 0.27 V reveals the corresponding deintercalation of K^+^ from the S-MCCF [24]. In the second cycle, a pair of broad cathodic/anodic peaks can be observed from 1.9–0.6 V/1.5–2.7 V, which corresponds to the reduction of doped-S in the S-MCCF and the reaction of doped-S with K^+^, respectively.

Figure 3b exhibits the charge/discharge profiles of the S-MCCF observed in the initial three cycles at a small current density of 50 mA g^−1^. It can be stated that the first discharge capacity is very high due to the following reasons: (1) a large interlayer distance can promote the intercalation of K^+^; (2) a small number of N, O, and S atoms might induce many active sites and defects, adsorbing K^+^ on the surface of the S-MCCF [28, 45]; (3) doped-S might reduce the adsorption energy during the potassiation process, which is beneficial to structural stability [27]; (4) a large specific surface area combined with many pores would provide a channel for electrolyte penetration and expedite the transport and adsorption of K^+^, with an increase in the pseudocapacitance [46–48]. Although the initial coulombic efficiency (ICE) is low due to the adsorption of a large amount of K^+^ on the surface and in the pores, which cannot retract, the charge/discharge curve of the third cycle nearly overlaps with that of the second cycle, demonstrating its high cycling stability from the second cycle.

Figure 3c, e displays the rate capability and long-cycling property of the S-MCCF, respectively. As indicated in Fig. 3c, after five activation cycles, the S-MCCF delivers high discharge capacities of 388.2, 332.1, 305.9, 262.5, 221.3, and 182.7 mAh g^−1^ at current densities of 50, 100, 200, 500, 1000, and 2000 mA g^−1^. When the current density is reset to 50 mAh g^−1^, the discharge capacity recovers to 310 mAh g^−1^, indicating super reversibility. The excellent rate performance is superior to that of many other carbonous anodes reported previously, as shown in Fig. 3d [5, 16, 20, 28, 46, 49–55]. The long-cycling performance of the S-MCCF was also evaluated at a high current density of 1000 mA g^−1^. In Fig. 3e, it can be observed that the S-MCCF electrode maintains a discharge capacity of ~ 150 mA g^−1^ with a high coulombic efficiency (CE) of ~ 100% after 2000 cycles, suggesting excellent cycling stability at high current densities.

To analyze the K^+^ storage behavior of the S-MCCF further, CV measurements were taken from 0.1 to 10 mV s^−1^, with the results presented in Fig. 3f. The sweep rate (*v*) and peak current (*i*) obey the following equation [56]:

4 $$i = av^{b}$$

In Eq. 4, *b *= 0.5 indicates a diffusion-dominated process (intercalation), while *b *= 1.0 represents a surface capacitive-dominated process, i.e., pseudo-capacitance K^+^ storage [10, 44]. Figure 3g shows the log(|*i*|)-log(*v*) curves and the calculated *b*-values. The *b*-values for the reduction and oxidation peaks are both 0.92, suggesting a surface capacitive-dominated process for K^+^ storage. Furthermore, the ratios of the capacitive contributions can be calculated based on the total current response (*i*) and scan rate (*v*), as shown in Eq. 5 [57, 58]:

5 $$i\left( V \right) = i_{\text{capacitive}} + i_{\text{diffusion}} = k_{1} v \, + \, k_{2} v^{1/2}$$

In Eq. 5, the adjustable parameters *k*_1_ and *k*_2_ can be determined by plotting *i*(*V*)/*v*^1/2^ versus *v*^1/2^ and the contributions of the surface capacitive and diffusion-dominated processes can subsequently be calculated by *k*_1_*v* and *k*_2_*v*^1/2^, respectively. Based on the results shown in Fig. 3h, the percentage of the capacitive-dominated contribution of the total specific capacity increased from 43.3% (0.1 mV s^−1^) to 88.7% (10 mV s^−1^) gradually, confirming that the storage mechanism predominantly involves the surface capacitive process in the S-MCCF anode.

### Electrochemical Performance of aMCCF Electrode

**Fig. 4:**
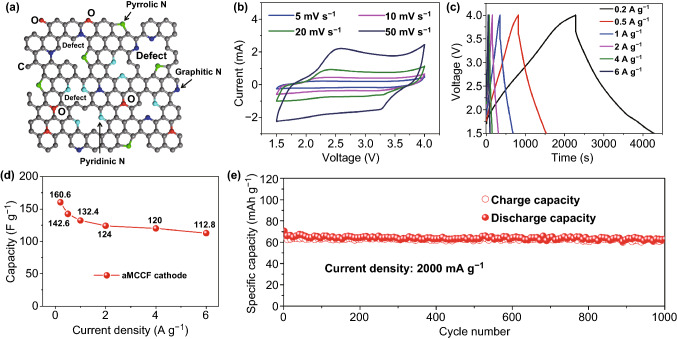
**a** Schematic showing the atomic structure of the aMCCF. The electrochemical performance of the aMCCF electrode with respect to the PIBs: **b** CV curves at a scan rate of 5–50 mV s^−1^, **c** Charge/discharge curves examined at a current density of 0.2–6 A g^−1^, **d** Plot of the capacity versus current density for the aMCCF, and **e** Long-term cycling property at 2000 mA g^−1^ (excluding the data of the first cycle)

In addition to the S-MCCF anode, the electrochemical behavior of the aMCCF cathode was also measured in the half-cell configuration versus K metal. Figure 4a exhibits a schematic of the atomic structure of the aMCCF, which is similar to that of the S-MCCF without S atom doping because these two structures are derived from the same precursor and undergo a similar preparation process. Compared with the S-MCCF, the aMCCF has a larger specific surface area and exhibit a mainly microporous structure (shown in Fig. 2c, d) due to KOH activation. The numerous hierarchical pores can provide more active sites and defects, facilitating the adsorption and transport of electrolyte ions during the fast charge/discharge process [52]. Therefore, the cyclic voltammetry (CV) curves at various scan rates for the aMCCF (shown in Fig. 4b) show a dominant electrochemical capacitive behavior, suggesting that the aMCCF is an ideal capacitor-type cathode material for PIHCs [10]. Furthermore, the capacities of the aMCCF were calculated based on the charge/discharge curves at serial current densities. The result shown in Fig. 4c, d demonstrates that the aMCCF electrodes have ultrafine electrochemical properties. To elaborate, the aMCCF can deliver high capacities of 160.6, 142.6, 132.4, 124, 120, and 112.8 F g^−1^ at a current density of 0.2–6 A g^−1^ in the KPF_6_ electrolyte (shown in Fig. 4d). Figure 4e shows the long-term cycling property of aMCCF at 2000 mA g^−1^. Clearly, there is almost no capacity decay over 1000 cycles, suggesting the excellent cycling stability of aMCCF at high current densities.

### Electrochemical Properties of PIHC Devices

**Fig. 5:**
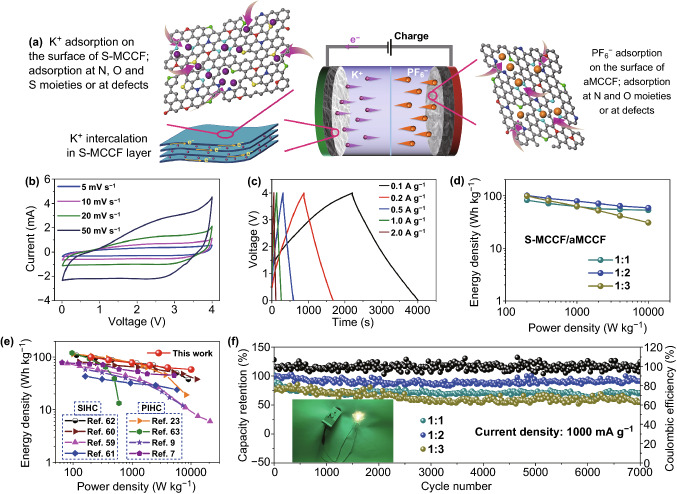
**a** Schematic illustration of the charge process for the S-MCCF//aMCCF PIHC. Performance of the PIHC (*m*_anode_:*m*_cathode_ = 1:2): **b** CV curves at different scan rates. **c** Charge/discharge curves at different current densities. **d** Ragone plots of the energy density versus power density for the PIHC with three mass ratios. **e** Ragone plots of the optimal S-MCCF//aMCCF PIHC (mass ratio of 1:2) compared with that of the reported SIHC and PIHC. **f** Cycling stability of the fabricated PIHC at 1000 mA g^−1^, with the inset showing a photo image of a LED panel powered by the PIHC

The excellent electrochemical performance of the S-MCCF and aMCCF electrodes demonstrates their potential practical application in PIHCs. Therefore, PIHC devices can be constructed with pre-activated S-MCCF as the anode. Figure 5a is a schematic illustration of the charge process for the PIHC. The K^+^ ion migrates to the anode and then intercalates into the carbon layer and absorbs onto the surface of the S-MCCF anode, while the anions in the electrolyte migrate and adsorb to the capacitor-type cathode surface. Else, the K^+^ ions and anions tend to migrate back into the electrolyte from the anode and cathode, respectively, during the discharge process. When aiming to fabricate the PIHC device ensuring both high energy and power density, the mass ratio of the S-MCCF anode to the aMCCF cathode should be optimized. Thus, three anode-to-cathode mass ratios of 1:1, 1:2, and 1:3 were evaluated in this experiment. The electrochemical performance of the PIHC was tested within a voltage range of 0.01–4 V.

Figure 5b, c and S5, S6 show the CV and galvanostatic charge/discharge curves (GCD) tested at scan rates of 5–50 mV s^−1^ and current densities ranging from 0.1 to 2 A g^−1^ (based on the total mass of the active electrodes). It can be seen that the CV curves slightly deviate from the desired rectangular shape, while the GCD slopes slightly deviate from the linear slope of a perfect supercapacitor, which confirms the combinatorial storage mechanism of the non-faradaic and faradaic reactions [10, 24, 59]. The energy and power densities of the PIHC with three mass ratios were calculated using Eqs. 2 and 3 (consult the Experimental section) based on the galvanostatic charge/discharge results and Ragone plots of the energy density versus power density, as shown in Fig. 5d. Following the calculations, the PIHC with an S-MCCF to aMCCF mass ratio of 1:2 exhibited the highest energy and power density. For example, the highest energy density of 100 Wh kg^−1^ can be delivered with a power density of 200 W kg^−1^. Besides, even at a higher power density of 10,000 W kg^−1^, the PIHC can still exhibit an energy density of 58.3 Wh kg^−1^. Further, the electrochemical performance of all the S-MCCF//aMCCF PIHCs is better than that of S-MCCF//MCCF PIHCs (shown in Fig. S7), confirming that the aMCCF material with large surface areas is an idea capacitor-type cathode for PIHCs and indicating that the KOH activation treatment is necessary for improving the electrochemical performance of PIHC devices. The excellent electrochemical performance of the S-MCCF//aMCCF (with a mass ratio of 1:2) PIHC is superior to that of many other Na-ion hybrid capacitors (SIHCs) and PIHCs reported previously, as shown in Fig. 5e [7, 9, 23, 59–63]. Furthermore, Fig. 5f shows that the PIHC (*m*_anode_:*m*_cathode_ = 1:2) achieved a capacity retention of ~ 90% over 7000 cycles with a CE of ~ 100% at 1 A g^−1^, demonstrating its long-cycling stability at high current densities. Moreover, this PIHC with the S-MCCF anode and aMCCF cathode (*m*_anode_:*m*_cathode_ = 1:2) can light up a light-emitting diode (LED) bulb, thus demonstrating its promising practical applications.

## Conclusion

In this study, we developed a “homologous strategy” to prepare carbonous electrode materials (S-MCCF anode and aMCCF cathode) for high-performance dual-carbon potassium-ion hybrid capacitors (PIHCs) by electrospinning the same precursor, followed by sulfur doping and KOH activation treatment, respectively. The S-MCCF anodes in the potassium-ion half-cells exhibited super cyclic stability and rate capability. The aMCCF cathodes showed a high specific surface area of 1445 m^2^ g^−1^ owing to its abundant multichannels and hierarchical pores. Thus, the aMCCF exhibited an excellent capacitive performance. Combining the merits of both the anode and cathode, the constructed S-MCCF//aMCCF PIHC (S-MCCF:aMCCF = 1:2, m:m) devices can deliver outstanding energy and power densities, which is superior to the properties of many previously reported SIHCs and PIHCs. Furthermore, the PIHC achieved a capacity retention of ~ 90% after 7000 cycles at 1 A g^−1^, demonstrating its long-cycling stability at high current densities. The excellent performance of PIHC devices can be ascribed to the following reasons: (1) the large interlayer distance combined with many pores of S-MCCF anode would promote the intercalation of K^+^ and expedite the transport and adsorption of K^+^; (2) a small number of heteroatoms in S-MCCF anode might induce many defects and active sites, adsorbing K^+^ on the S-MCCF surface; (3) aMCCF cathode with large specific surface area is suitable to match the S-MCCF anode; (4) the similar chemical stability of the electrodes can improve the cycling stability. Our proposed synthetic method, combined with the distinctive electrochemical performance of dual-carbon PIHCs, might provide a new method for the fabrication of high-performance alkali-ion batteries and capacitors, with a speed-up of their practical applications.

## Electronic Supplementary Material

Below is the link to the electronic supplementary material.

Supplementary material 1 (PDF 1439 kb)

## References

[1] Ren X, Ren Z, Li Q, Wen W, Li X (2019). Tailored plum pudding-like Co_2_P/Sn encapsulated with carbon nanobox shell as superior anode materials for high-performance sodium-ion capacitors. Adv. Energy Mater..

[2] Su D, McDonagh A, Qiao S-Z, Wang G (2017). High-capacity aqueous potassium-ion batteries for large-scale energy storage. Adv. Mater..

[3] Xu J, Li Y, Wang L, Cai Q, Li Q (2016). High-energy lithium-ion hybrid supercapacitors composed of hierarchical urchin-like WO_3_/C anodes and MOF-derived polyhedral hollow carbon cathodes. Nanoscale.

[4] Zhao Y, Ruan J, Luo S, Sun H, Pang Y, Yang J, Zheng S (2019). Rational construction of a binder-free and universal electrode for stable and fast alkali-ion storage. ACS Appl. Mater. Interfaces.

[5] Wang W, Zhou J, Wang Z, Zhao L, Li P (2018). Short-range order in mesoporous carbon boosts potassium-ion battery performance. Adv. Energy Mater..

[6] Xiong P, Bai P, Tu S, Cheng M, Zhang J, Sun J, Xu Y (2018). Red phosphorus nanoparticle@3D interconnected carbon nanosheet framework composite for potassium-ion battery anodes. Small.

[7] Zhang Z, Li M, Gao Y, Wei Z, Zhang M (2018). Fast potassium storage in hierarchical Ca_0.5_Ti_2_(PO_4_)_3_@C microspheres enabling high-performance potassium-ion capacitors. Adv. Funct. Mater..

[8] Zhou L, Zhang M, Wang Y, Zhu Y, Fu L (2017). Cubic Prussian blue crystals from a facile one-step synthesis as positive electrode material for superior potassium-ion capacitors. Electrochim. Acta.

[9] Dong S, Li Z, Xing Z, Wu X, Ji X, Zhang X (2018). Novel potassium-ion hybrid capacitor based on an anode of K_2_Ti_6_O_13_ microscaffolds. ACS Appl. Mater. Interfaces..

[10] Chen J, Yang B, Hou H, Li H, Liu L, Zhang L, Yan X (2019). Disordered, large interlayer spacing, and oxygen-rich carbon nanosheets for potassium ion hybrid capacitor. Adv. Energy Mater..

[11] Chen J, Yang B, Li H, Ma P, Lang J, Yan X (2019). Candle soot: onion-like carbon, an advanced anode material for a potassium-ion hybrid capacitor. J. Mater. Chem. A.

[12] Li D, Ren X, Ai Q, Sun Q, Zhu L (2018). Facile fabrication of nitrogen-doped porous carbon as superior anode material for potassium-ion batteries. Adv. Energy Mater..

[13] Liu C, Xiao N, Li H, Dong Q, Wang Y (2020). Nitrogen-doped soft carbon frameworks built of well-interconnected nanocapsules enabling a superior potassium-ion batteries anode. Chem. Eng. J..

[14] Ruan J, Zhao Y, Luo S, Yuan T, Yang J, Sun D, Zheng S (2019). Fast and stable potassium-ion storage achieved by in situ molecular self-assembling N/O dual-doped carbon network. Energy Storage Mater..

[15] Tao L, Yang Y, Wang H, Zheng Y, Hao H (2020). Sulfur-nitrogen rich carbon as stable high capacity potassium ion battery anode: performance and storage mechanisms. Energy Storage Mater..

[16] Qi X, Huang K, Wu X, Zhao W, Wang H, Zhuang Q, Ju Z (2018). Novel fabrication of N-doped hierarchically porous carbon with exceptional potassium storage properties. Carbon.

[17] Sun Y, Xiao H, Li H, He Y, Zhang Y (2019). Nitrogen/oxygen co-doped hierarchically porous carbon for high-performance potassium storage. Chem. Eur. J..

[18] Xiong P, Zhao X, Xu Y (2018). Nitrogen-doped carbon nanotubes derived from metal-organic frameworks for potassium-ion battery anodes. Chemsuschem.

[19] Share K, Cohn AP, Carter R, Rogers B, Pint CL (2016). Role of nitrogen doped graphene for improved high capacity potassium ion battery anodes. ACS Nano.

[20] Xu Y, Zhang C, Zhou M, Fu Q, Zhao C, Wu M, Lei Y (2018). Highly nitrogen doped carbon nanofibers with superior rate capability and cyclability for potassium ion batteries. Nat. Commun..

[21] Gong S, Wang Q (2017). Boron-doped graphene as a promising anode material for potassium-ion batteries with a large capacity, high rate performance, and good cycling stability. J. Phys. Chem. C.

[22] Ma G, Huang K, Ma J-S, Ju Z, Xing Z, Zhuang Q (2017). Phosphorus and oxygen dual-doped graphene as superior anode material for room-temperature potassium-ion batteries. J. Mater. Chem. A.

[23] Qiu D, Guan J, Li M, Kang C, Wei J (2019). Kinetics enhanced nitrogen-doped hierarchical porous hollow carbon spheres boosting advanced potassium-ion hybrid capacitors. Adv. Funct. Mater..

[24] Yang B, Chen J, Liu L, Ma P, Liu B (2019). 3D nitrogen-doped framework carbon for high-performance potassium ion hybrid capacitor. Energy Storage Mater..

[25] Xu D, Chen C, Xie J, Zhang B, Miao L (2016). A hierarchical N/S-codoped carbon anode fabricated facilely from cellulose/polyaniline microspheres for high-performance sodium-ion batteries. Adv. Energy Mater..

[26] Qie L, Chen W, Xiong X, Hu C, Zou F, Hu P, Huang Y (2015). Sulfur-doped carbon with enlarged interlayer distance as a high-performance anode material for sodium-ion batteries. Adv. Sci..

[27] Li J, Qin W, Xie J, Lei H, Zhu Y (2018). Sulphur-doped reduced graphene oxide sponges as high-performance free-standing anodes for K-ion storage. Nano Energy.

[28] Chen M, Wang W, Liang X, Gong S, Liu J (2018). Sulfur/oxygen codoped porous hard carbon microspheres for high-performance potassium-ion batteries. Adv. Energy Mater..

[29] Ding J, Zhang H, Zhou H, Feng J, Zheng X (2019). Sulfur-grafted hollow carbon spheres for potassium-ion battery anodes. Adv. Mater..

[30] Tian S, Guan D, Lu J, Zhang Y, Liu T (2020). Synthesis of the electrochemically stable sulfur-doped bamboo charcoal as the anode material of potassium-ion batteries. J. Power Sources.

[31] Hou R, Liu B, Sun Y, Liu L, Meng J (2020). Recent advances in dual-carbon based electrochemical energy storage devices. Nano Energy.

[32] Chen J, Yang B, Liu B, Lang J, Yan X (2019). Recent advances in anode materials for sodium- and potassium-ion hybrid capacitors. Curr. Opin. Electrochem..

[33] Xu Y, Yuan T, Zhao Y, Yao H, Yang J, Zheng S (2019). Constructing multichannel carbon fibers as freestanding anodes for potassium-ion battery with high capacity and long cycle life. Adv. Mater. Interfaces.

[34] Park S-H, Jung H-R, Lee W-J (2013). Hollow activated carbon nanofibers prepared by electrospinning as counter electrodes for dye-sensitized solar cells. Electrochim. Acta.

[35] Zhou Y, He J, Wang H, Qi K, Ding B, Cui S (2016). Carbon nanofiber yarns fabricated from co-electrospun nanofibers. Mater. Design.

[36] Kaerkitcha N, Chuangchote S, Sagawa T (2016). Control of physical properties of carbon nanofibers obtained from coaxial electrospinning of PMMA and PAN with adjustable inner/outer nozzle-ends. Nanoscale Res. Lett..

[37] Zhang B, Kang F, Tarascon J-M, Kim J-K (2016). Recent advances in electrospun carbon nanofibers and their application in electrochemical energy storage. Prog. Mater Sci..

[38] Shi R, Han C, Xu X, Qin X, Xu L (2018). Electrospun N-doped hierarchical porous carbon nanofiber with improved degree of graphitization for high-performance lithium ion capacitor. Chem. Eur. J..

[39] Yang ZY, Wang YH, Dai Z, Lu ZW, Gu XY (2019). Nature of improved double-layer capacitance by KOH activation on carbon nanotube-carbon nanofiber hierarchical hybrids. Carbon.

[40] Yang J, Ju Z, Jiang Y, Xing Z, Xi B, Feng J, Xiong S (2018). Enhanced capacity and rate capability of nitrogen/oxygen dual-doped hard carbon in capacitive potassium-ion storage. Adv. Mater..

[41] Yuan C, Liu X, Jia M, Luo Z, Yao J (2015). Facile preparation of N- and O- doped hollow carbon spheres derived from poly(o-phenylenediamine) for supercapacitors. J. Mater. Chem. A.

[42] Ju Z, Li P, Ma G, Xing Z, Zhuang Q, Qian Y (2018). Few layer nitrogen-doped graphene with highly reversible potassium storage. Energy Storage Mater..

[43] Yang J, Zhou X, Wu D, Zhao X, Zhou Z (2016). S-doped N-rich carbon nanosheets with expanded interlayer distance as anode materials for sodium-ion batteries. Adv. Mater..

[44] Adams RA, Syu J-M, Zhao Y, Lo C-T, Varma A, Pol VG (2017). Binder-free N- and O- rich carbon nanofiber anodes for long cycle life K-ion batteries. ACS Appl. Mater. Interfaces.

[45] Shao W, Hu F, Song C, Wang J, Liu C, Weng Z, Jian X (2019). Hierarchical N/S co-doped carbon anodes fabricated through facile ionothermal polymerization for high-performance sodium ion batteries. J. Mater. Chem. A.

[46] Liu L, Chen Y, Xie Y, Tao P, Li Q, Yan C (2018). Understanding of the ultrastable K-ion storage of carbonaceous anode. Adv. Funct. Mater..

[47] Huang K, Xing Z, Wang L, Wu X, Zhao W (2017). Direct synthesis of 3D hierarchically porous carbon/Sn composites via in situ generated NaCl crystals as templates for potassium-ion batteries anode. J. Mater. Chem. A.

[48] Zhao X, Xiong P, Meng J, Liang Y, Wang J, Xu Y (2017). High rate and long cycle life porous carbon nanofiber paper anodes for potassium-ion batteries. J. Mater. Chem. A.

[49] Mahmood A, Li S, Ali Z, Tabassum H, Zhu B (2019). Ultrafast sodium/potassium-ion intercalation into hierarchically porous thin carbon shells. Adv. Mater..

[50] Xie Y, Chen Y, Liu L, Tao P, Fan M (2017). Ultra-high pyridinic N-doped porous carbon monolith enabling high-capacity K-ion battery anodes for both half-cell and full-cell applications. Adv. Mater..

[51] Tai Z, Zhang Q, Liu Y, Liu H, Dou S (2017). Activated carbon from the graphite with increased rate capability for the potassium ion battery. Carbon.

[52] Lu G, Wang H, Zheng Y, Zhang H, Yang Y (2019). Metal-organic framework derived N-doped CNT@ porous carbon for high-performance sodium- and potassium-ion storage. Electrochim. Acta.

[53] Cao W, Zhang E, Wang J, Liu Z, Ge J (2019). Potato derived biomass porous carbon as anode for potassium ion batteries. Electrochim. Acta.

[54] Gao C, Wang Q, Luo S, Wang Z, Zhang Y (2019). High performance potassium-ion battery anode based on biomorphic N-doped carbon derived from walnut septum. J. Power Sources.

[55] Li H, Cheng Z, Zhang Q, Natan A, Yang Y, Cao D, Zhu H (2018). Bacterial-derived, compressible, and hierarchical porous carbon for high-performance potassium-ion batteries. Nano Lett..

[56] Ruan J, Mo F, Chen Z, Liu M, Zheng S (2020). Rational construction of nitrogen-doped hierarchical dual-carbon for advanced potassium-ion hybrid capacitors. Adv. Energy Mater..

[57] Luo D, Xu J, Guo Q, Fang L, Zhu X, Xia Q, Xia H (2018). Surface-dominated sodium storage towards high capacity and ultrastable anode material for sodium-ion batteries. Adv. Funct. Mater..

[58] Liu J, Wang J, Xu C, Jiang H, Li C (2018). Advanced energy storage devices: basic principles, analytical methods, and rational materials design. Adv. Sci..

[59] Lim E, Jo C, Kim MS, Kim M-H, Chun J (2016). High-performance sodium-ion hybrid supercapacitor based on Nb_2_O_5_@carbon core-shell nanoparticles and reduced graphene oxide nanocomposites. Adv. Funct. Mater..

[60] Zhu Y-E, Yang L, Sheng J, Chen Y, Gu H, Wei J, Zhou Z (2017). Fast sodium storage in TiO_2_@CNT@C nanorods for high-performance Na-ion capacitors. Adv. Energy Mater..

[61] Li H, Zhu Y, Dong S, Shen L, Chen Z, Zhang X, Yu G (2016). Self-assembled Nb_2_O_5_ nanosheets for high energy-high power sodium ion capacitors. Chem. Mater..

[62] Li D, Ye C, Chen X, Wang S, Wang H (2018). A high energy and power sodium-ion hybrid capacitor based on nitrogen-doped hollow carbon nanowires anode. J. Power Sources.

[63] Fan L, Lin K, Wang J, Ma R, Lu B (2018). A nonaqueous potassium-based battery-supercapacitor hybrid device. Adv. Mater..

