# Supplementary material for: Homologous Strategy to Construct High-Performance Coupling Electrodes for Advanced Potassium-Ion Hybrid Capacitors
Source: Nanomicro Lett. 2020 Oct 30;13:14. doi: 10.1007/s40820-020-00524-z (PMC8187694; doi:10.1007/s40820-020-00524-z)
Supplement: Supplementary file 1 — Supplementary material 1 (PDF 1439 kb) [file 40820_2020_524_MOESM1_ESM.pdf]

Supporting Information for

Homologous Strategy to Construct High-Performance Coupling Electrodes for Advanced Potassium-Ion Hybrid Capacitors

Ying Xu[1], Jiafeng Ruan[1], Yuepeng Pang[1], Hao Sun[1], Chu Liang[2,] *, Haiwen Li[3], Junhe Yang[1,] *, Shiyou Zheng[1,] *

[1]School of Materials Science and Engineering, University of Shanghai for Science and Technology, Shanghai 200093, People's Republic of China

[2]College of Materials Science and Engineering, Zhejiang University of Technology, Hangzhou 310014, People's Republic of China

[3]International Research Center for Hydrogen Energy, Kyushu University, Fukuoka 819-0395, Japan

*Corresponding authors. E-mail: cliang@zjut.edu.cn (C. Liang); jhyang@usst.edu.cn (J. Yang); syzheng@usst.edu.cn (S. Zheng)

Supplementary Figures

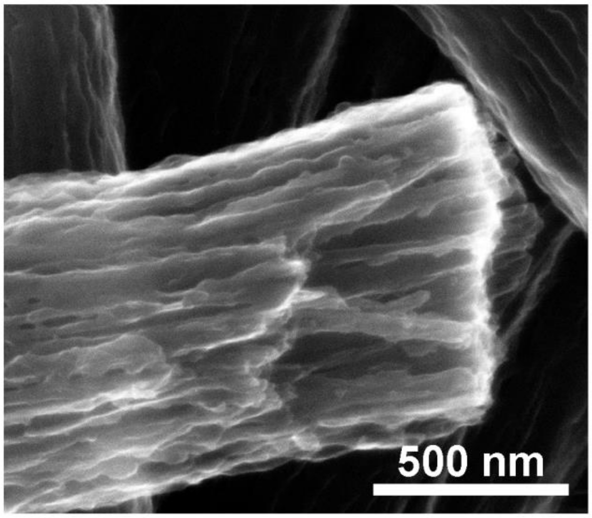

Fig. S1 SEM imagine of MCCF

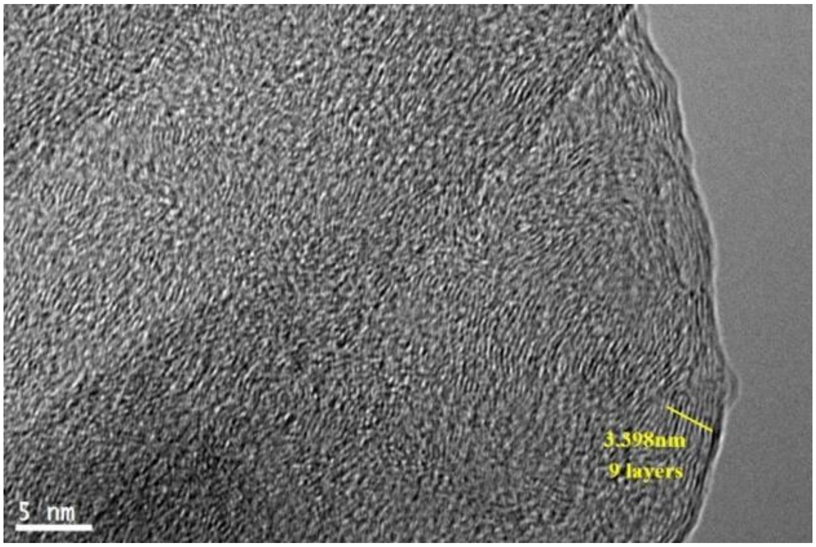

Fig. S2 TEM imagine with larger magnification of S-MCCF, in which the interlayer distance calculated to be about 0.38 nm

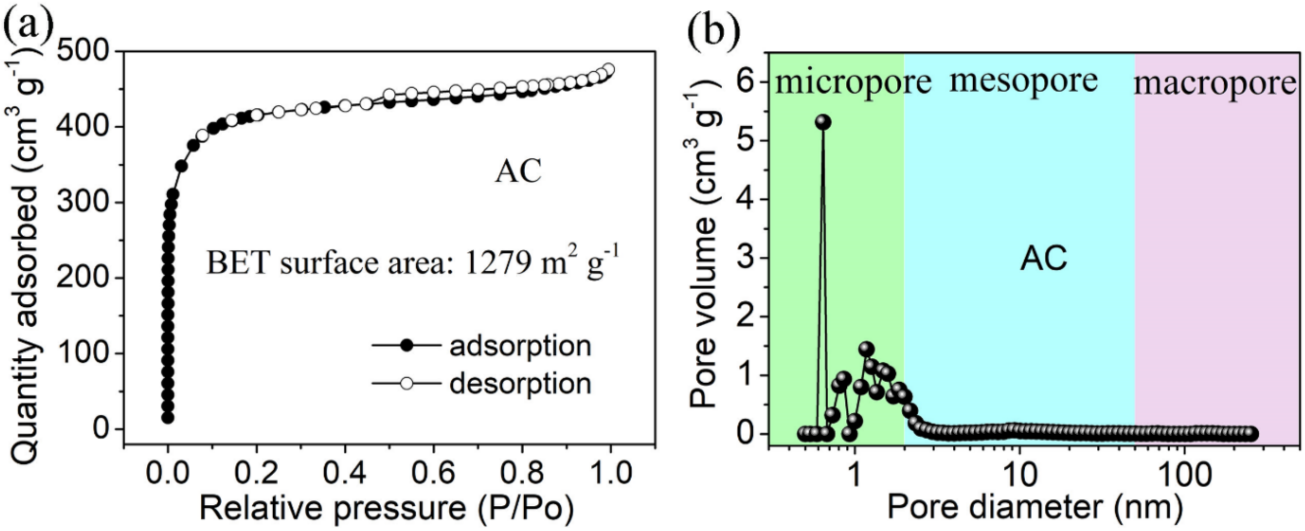

Fig. S3 a N_2 adsorption-desorption isothermal curves and **b** Pore size distributions of AC

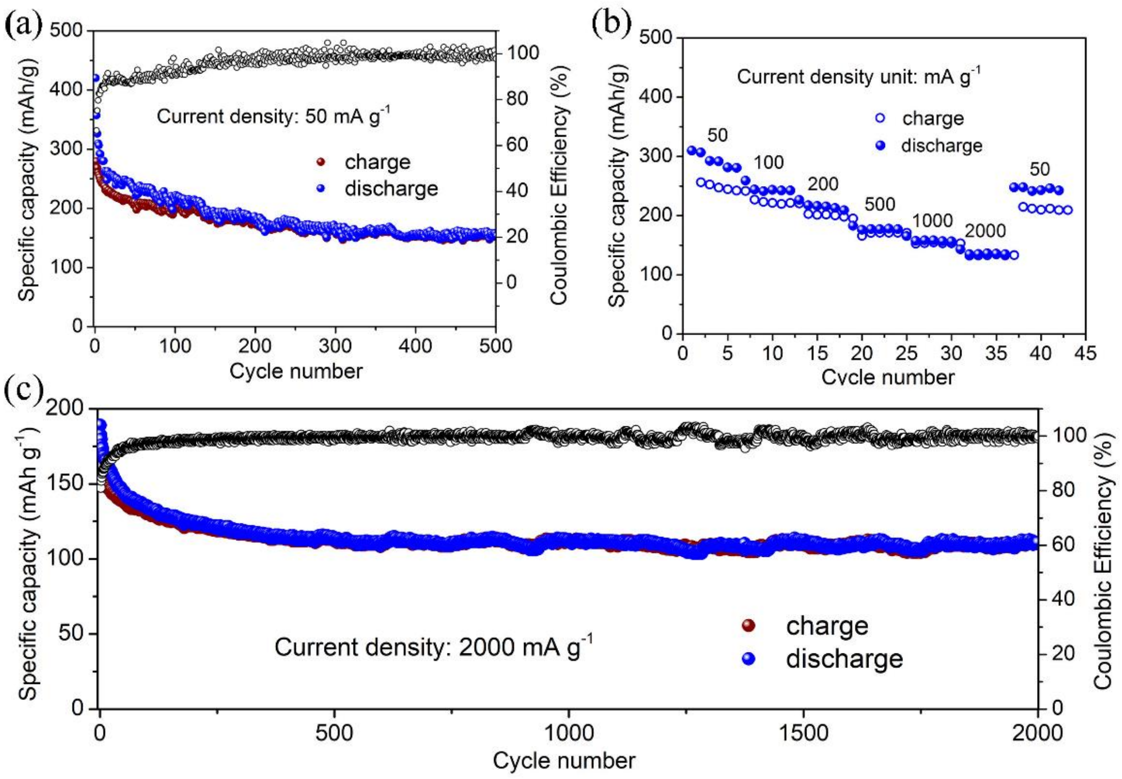

Fig. S4 Electrochemical performance of MCCF anode for PIBs: **a** Cycling performance at the current density of 50 mA g^{-1}. **b** Rate performances tested at 50, 100, 200, 500, 1000, 2000, and 50 mA g^{-1}. **c** Long-term cycling performance at a current density of 2000 mA g^{-1}

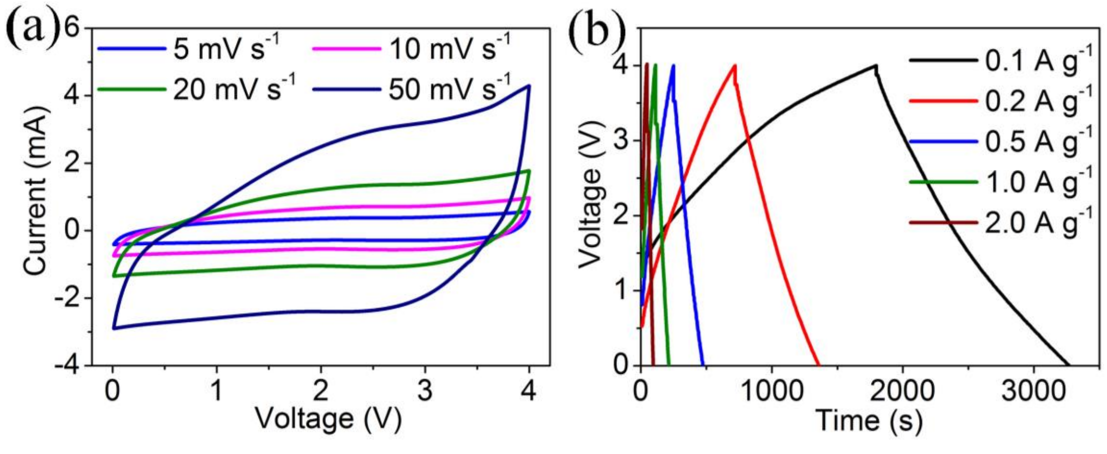

Fig. S5 **a** CV curves and **b** GCD profiles of S-MCCF//aMCCF PIHC with an anode to cathode mass ratio of 1:1

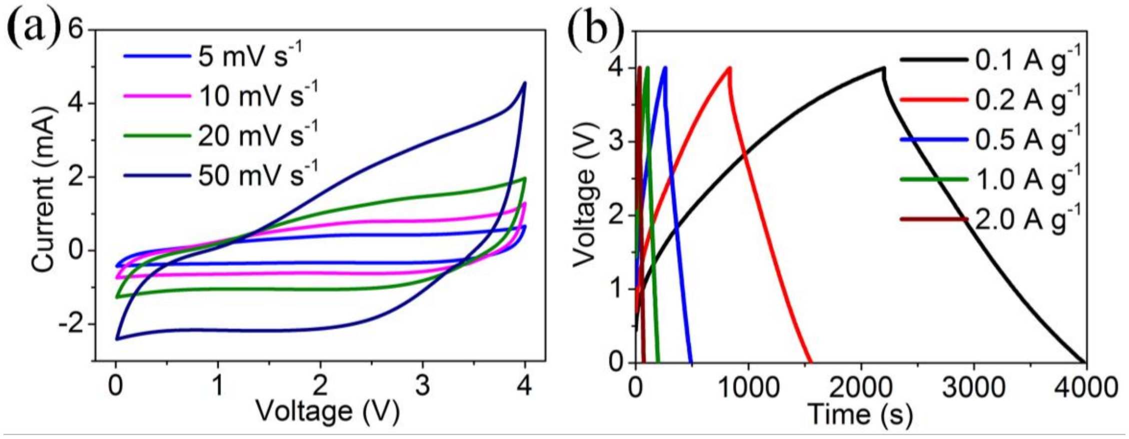

Fig. S6 a CV curves and **b** GCD profiles of S-MCCF//aMCCF PIHC with an anode to cathode mass ratio of 1:3

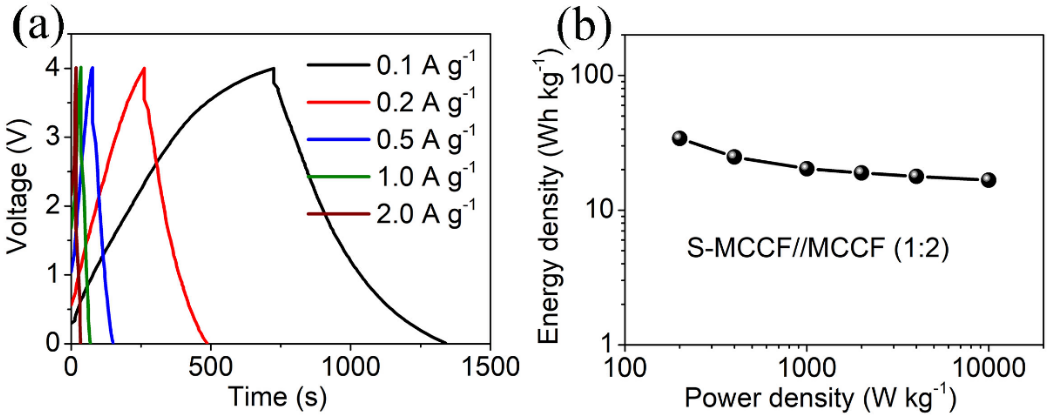

Fig. S7 a GCD profiles and **b** Ragone plots of the energy density versus power density for S-MCCF//MCCF PIHC with an anode to cathode mass ratio of 1:2
